# Benefits of organic manure combined with biochar amendments to cotton root growth and yield under continuous cropping systems in Xinjiang, China

**DOI:** 10.1038/s41598-020-61118-8

**Published:** 2020-03-13

**Authors:** Zhiyong Zhang, Xiuxiu Dong, Shaoming Wang, Xiaozhen Pu

**Affiliations:** 1 College of Life Sciences, Shihezi University, Shihezi, 832003 China; 2 Pharmacy School, Shihezi University/Key Laboratory of Xinjiang Phytomedicine Resource and Utilization, Ministry of Education, Shihezi, 832003 China

**Keywords:** Field trials, Biophysical chemistry

## Abstract

Organic manure and biochar amendments have been used in agriculture to improve soil fertility and enhance crop productivity. Plant roots play an important role in the functionality of individual plants, and although the addition of organic manure and biochar reportedly affect roots, it remains unclear how root morphology and physiology respond. We conducted a field experiment to test the hypothesis that organic manure combined with biochar amendment could also enhance the productivity of continuous cropping systems in Xinjiang cotton plantations. Different levels of organic manure and biochar were applied. Organic manure and biochar significantly affected root morphology and physiology by improving soil nutrients. In the absence of biochar, organic manure amendment increased Root TTC reducing capacity, glutamine synthetase and nitrate reductase activity. Furthermore, morphological and physiological parameters peaked with 6% organic manure combined with 1% biochar. A significant increase in root physiology was recognized with an increase in soil nutrient content at the bud stage and a negative relationship between root physiology and soil total K content at the harvesting stage. Thus, our results indicate that organic manure combined with biochar positively influenced cotton roots, and therefore should be used to improve root health in continuous cropping systems.

## Introduction

Xinjiang has abundant land resources, and its climate has low precipitation, high levels of sunshine and large temperature difference between day and night. When combined with the large-scale application of drip irrigation and mechanized cotton planting, this ensures the higher economic benefit of cotton in Xinjiang compared with other inland cotton producing areas. Compared with other crops such as safflower, maize, and wheat, cotton has outstanding advantages and stable benefits including high profit, advantage of planting technology and preferential policy^1^. By 2016, the cotton planting area and cotton yield in Xinjiang accounted for 53.97% and 67.3% of the whole country, respectively. Owing to the slow process of agricultural industrialization, large area of the land involved, short period of contracts, and driven by economic interests, continuous cropping is widespread in Xinjiang^2^. 73.1% of cotton fields in Xinjiang have been continuously cropped for more than five years^3^.

In continuous cropping systems, demand for nutrients causes imbalances in soil nutrients in cotton fields, affects the growth, nutrient uptake, and utilization efficiency of roots, and ultimately reduces yield. Previous studies have reported a significant correlation between cotton yield and soil N, C, and P quality ratios during continuous cropping^4^. Soil organic matter, available P, and available K, and trace elements, including available Mn, available Fe, and available Cu exhibit different trends and degrees of change, which leads to imbalances in soil nutrients in cotton fields^5^. In addition, the yield of cotton fields continuously cropped for the same number of years was correlated with soil nutrient content. Soil organic matter and available N content were higher in high-yielding fields. However, there was no significant difference between available P and available K^6^. In addition, owing to the deterioration of the soil environment, continuous cropping leads to the frequent occurrence of cotton pests and diseases, such as cotton aphids, cotton bollworms, Verticillium wilt, and cotton root rot^7,8^.

Animal manures have been widely used as fertilizers for centuries. Organic fertilizers contain not only the basic nutrients required by crops but also trace elements. Chicken manure is rich in mineral elements, essential nutrients N, P, and K, and other nutrients that can improve soil physical and chemical properties^9^. Organic matter is an important component of organic fertilizers, and organic acids contained in organic matter can acidify nutrients improving their effectiveness^10^. In addition to chemical fertilizers, manure is an important source of nutrients for agricultural production in China. Application of manure helps maintain soil nutrient balance, improves soil structure, and moisture-holding capacity, and is beneficial for environmental protection compared with the application of chemical fertilizers alone^11^. As a result of organic amendments, heterogeneity in resource distribution is likely to arise, and the subsequent microbial decomposition of both simple and complex organic materials will release organic and inorganic N for plant uptake. The spatial and temporal release of these nutrients will be more complex than when inorganic nutrients are applied directly^12^. Tian *et al*.^13^ reported that organic fertilizers significantly increased soil organic matter content and active components and promoted nutrient absorption based on 3-year cotton pot experiments. Manure incorporation into subsoil increased transpiration efficiency, and resulted in a doubling of root length density and root surface area in the manured layer^14^. Organic amendment (chicken manure) improved first order lateral root number, tap root length, fine root morphology (length, surface area, and volume) in seedlings, organic fertilizer treatments tended to increase soil ammonium, nitrate, available P, total P, and organic C content^15^. Similarly, owing to the high nutrient content, organic fertilizer cannot be fully absorbed and utilized by crops, resulting in nutrient leaching and rapid nutrient release, which cannot guarantee to meet the nutrient demands of crops throughout the growth period. In addition, feed additive use in livestock and poultry leads to excessive content of trace elements such as Cu, Fe, Zn, Mn, Co, Se, I, and As in livestock and poultry manure, as well as persistent organic pollutants such as polycyclic aromatic hydrocarbons and organochlorines^16^. Long-term application of this kind of organic fertilizer will lead to heavy metal pollution in soil and affect the growth and quality of crops.

As a soil amendment agent, biochar has unique physical and chemical characteristics. By improving soil physical structure, optimizing microbial growth and reproduction, reducing soil nutrient leaching, and reducing the occurrence of pests and diseases, biochar can promote crop growth, development, yield, and quality^17–19^. Biochar can improve soil physical and chemical properties, reduce nutrient leaching, optimize soil microbial population structure and richness, and promote the growth and function of crop roots^20^. Adding biochar reduced soil compactness and affected soil water holding capacity and microbial and root growth^21^. Plants grown in biochar-amended soil had greater root mass and root length densities, and total and individual root lengths for secondary and tertiary roots compared with plants grown in unamended soil^22^.

The results of Zhang *et al*.^23^ showed that the addition of corn straw biochar could promote the growth of the rice root system in sandy loam soil, increase root volume, total and active absorption area, and maintain high Root TTC reducing capacity. In addition, biochar indirectly improved soil fertility mainly by affecting the soil environment, and the main nutrients in biochar exist in the form of biological solids, and the effectiveness of nutrients depends on the biomineralization process, resulting in slow nutrient supply from biochar^24^. In addition, biochar has strong adsorption functions, which can effectively adsorb toxic substances produced by continuous cropping and heavy metals from organic fertilizer application^25^.

Biochar combined with organic fertilizer not only effectively alleviates the problems of excessive fertilizer use such as environmental pollution but also activates soil microorganisms and soil enzymes^26^, improving the potential value of biochar as a soil amendment. In the present study, we focused on the effects of organic fertilizer with and without biochar addition on soil and cotton root system properties in a cotton field under continuous cropping in Xinjiang. Our aim was to provide a theoretical basis for further developing cotton continuous cropping in Xinjiang. Our hypothesis was that organic fertilizer both with and without biochar addition can improve soil nutrients, cotton root growth, and biomass accumulation but that the addition of biochar would results in more improved properties.

## Results

### Effect of organic manure and combined with biochar on root morphology

**Figure 1:**
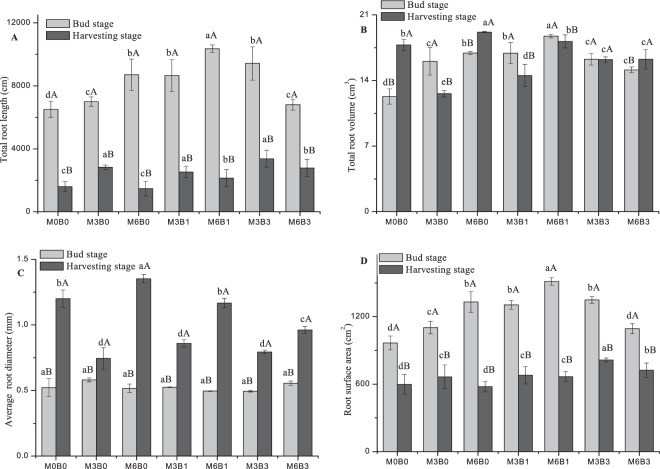
Changes in total root length, total root volume, average diameter, and root surface area of cotton under different treatments. M represents organic manure levels (M3, M6); B represents biochar levels (B1, B3). Different uppercase and lowercase letters indicate a significant difference between different growth stages and different organic manure combined with biochar rates, respectively, at P < 0.05. Error bars represent the standard error of the mean.

The total root length and root surface area were higher in the bud stage than the harvesting stage (Fig. 1A,D). In contrast, the average root diameter was higher in the harvesting stage than the bud stage (Fig. 1C). Total root volume was the highest in the harvesting stage, except in M0B0, M6B0, and M6B3 (Fig. 1B). In the absence of biochar, organic manure application significantly increased total root length, total root volume, and root surface area in the bud and harvesting stages compared with the control (no organic manure and biochar). However, organic manure had no significant effect on average root diameter in the bud stage. The total root length, total root volume, and root surface area were the highest when treated with 6% organic manure.

Organic manure combined with biochar significantly increased total root length compared with no biochar (Fig. 1A). Similarly, 3% organic manure combined with biochar significantly increased root surface area in the bud stage but the change was not significant in the harvesting stage (Fig. 1D). With 6% organic manure combined with biochar, the root surface area in the bud stage was in the following order: M6B1 > M6B0 > M6B3. However, in the harvesting stage the order was: M6B3 > M6B1 > M6B0. Furthermore, the average root diameter decreased with 6% organic manure and biochar (Fig. 1C). The total root length, total root volume, and root surface area were the highest when treated M6B1 (Fig. 1A,B,D).

**Figure 2:**
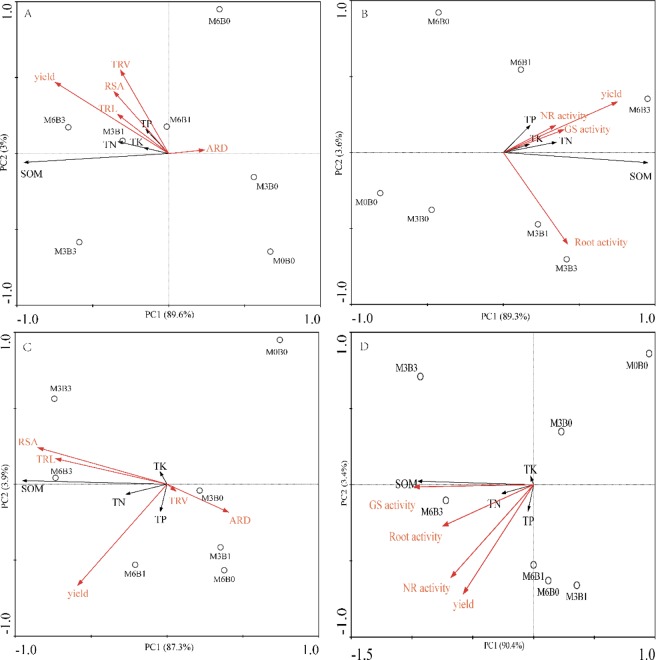
Effect of organic manure and combined with biochar amendments on root growth and soil nutrients at bud stage (**A,B**), harvesting stage (**C,D**); with root morphology (**A,C**) and with root physiology (**B,D**).

The application of organic fertilizer significantly increased the content of soil organic matter, total N, and total P in the bud and harvesting stages. The application of organic fertilizer significantly increased total K content of soil in the bud stage and decreased total K content of soil in the harvesting stage. The organic matter, total N, total P, and total K content of soils in the bud and harvesting stages were significantly increased by the combination of two levels of organic manure and biochar compared with those without biochar (Table 1). The correlation analysis showed that the total root length, root volume, and root surface area of cotton roots were positively correlated with soil nutrients. However, average diameter was negatively correlated with soil nutrients (Fig. 2).

**Table 1:** Changes in soil nutrients under different treatments.

Treatments	Bud stage				Harvesting stage			
	SOM (g kg^−1^)	TN (g kg^−1^)	TP (g kg^−1^)	TK (g kg^−1^)	SOM (g kg^−1^)	TN (g kg^−1^)	TP (g kg^−1^)	TK (g kg^−1^)
M0B0	28.42e	1.19c	1.34c	33.61c	38.28c	1.21c	0.89c	35.04a
M3B0	30.64d	1.38c	1.53b	33.85c	54.09b	1.58b	1.16b	31.35b
M6B0	34.16d	1.66b	1.96a	36.74b	58.74b	1.62b	1.30a	30.00b
M3B1	47.76c	1.66b	1.56b	35.77b	57.07b	1.59b	1.38a	34.32a
M6B1	50.94c	1.77b	2.04a	38.35b	62.30b	1.82b	1.44a	33.20a
M3B3	75.88b	2.09a	1.78b	39.66a	124.29a	2.02a	1.10b	35.08a
M6B3	81.55a	2.46a	2.15a	41.84a	133.67a	2.31a	1.23b	34.80a

Note: SOM: soil organic matter. The value labeled with different letters in the same column are significantly different among treatments at 0.05 level. M stands for organic manure levels (M3, M6); B stands for biochar levels (B1, B3).

### Effect of organic manure and combined with biochar on root physiology

**Figure 3:**
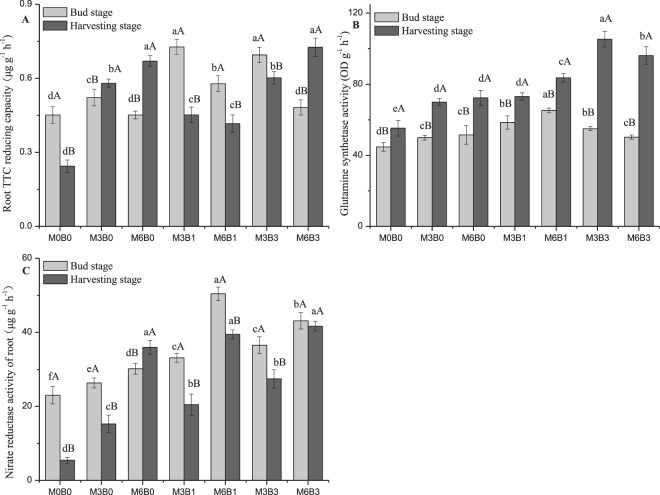
Changes in root, GS, and NR activity of cotton under different treatments. M represents organic manure levels (M3, M6); B represents biochar levels (B1, B3). Different uppercase and lowercase letters indicate a significant difference between different growth stages and different organic manure combined with biochar rates, respectively, at P < 0.05. Error bars represent the standard error of the mean.

The highest Root TTC reducing capacity was in the bud stage except in M3B0, M6B0, and M6B3 (Fig. 3A). The highest nitrate reductase (NR) activity was in the bud stage except in M6B0 (Fig. 3C). However, the highest glutamine synthetase (GS) activity was in the harvesting stage (Fig. 3B). In the absence of biochar, organic manure application significantly increased Root TTC reducing capacity and GS activity in the two growth stages, which were at their highest when treated with 6% organic manure.

The highest root, GS, and NR activity were observed under treatments with organic manure combined with 1% biochar in the bud stage but with 3% biochar in the harvesting stage (Fig. 3A–C). Furthermore, the Root TTC reducing capacity was higher in treatments with 3% organic manure than 6% organic manure in the bud stage but opposite in the harvesting stage. However, GS activity was higher in treatments with 6% organic manure than 3% organic manure in the bud stage and opposite in the harvesting (Fig. 3B). The highest NR activity was in treatments with 6% organic manure in the bud and harvesting stages (Fig. 3C). Cotton root, GS, and NR activity were positively correlated with soil nutrients. In addition, cotton Root TTC reducing capacity, GS and NR activity were positively correlated with soil total K in the bud stage and negatively correlated with soil total K in the harvesting stage (Fig. 2).

### Effect of organic manure and combined with biochar on dry matter accumulation of cotton

**Figure 4:**
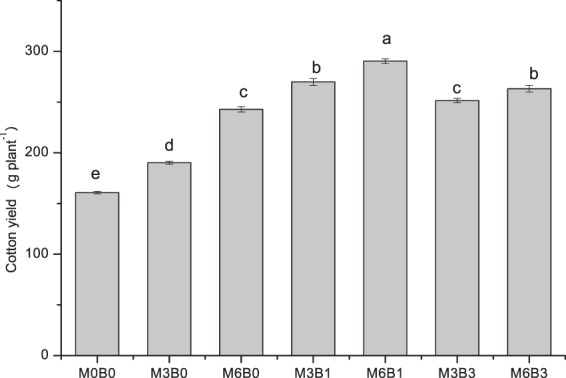
Organic manure (M) with or without biochar (B) effects on yield of cotton. Different letters indicate significant differences at P < 0.05. Error bars represent the standard error of the mean.

Application of organic fertilizer significantly increased the underground and aboveground biomass of cotton in the bud and harvesting stages (Table 2). In bud stage, organic fertilizer combined with biochar increase properties in the following order: 1% > 3% > M0B0. In the harvesting stage, biomass was significantly increased with the additional application of biochar. However, there was no significant difference between the two levels of biochar additions. Individual application of organic fertilizer significantly increased cotton yield per plant but increased further with biochar addition (Fig. 4). Yield increase was higher with organic fertilizer combined with 1% biochar than that with 3% biochar. In addition, 6% organic fertilizer was more effective than 3% organic fertilizer. Thus, cotton yield per plant was the highest under M6B1 treatment.

**Table 2:** Organic manure (M) with or without biochar (B) effects on dry matter accumulation of cotton.

Treatments	Bud stage			Harvesting stage		
	Root dry weight (g plant^−1^)	Shoot dry weight (g plant^−1^)	Root−shoot ratio	Root dry weight (g plant^−1^)	Shoot dry weight (g plant^−1^)	Root-shoot ratio
M0B0	20.383c	76.290d	0.268b	35.550c	355.907c	0.099a
M3B0	20.423c	88.140c	0.233c	41.015b	463.117b	0.089b
M6B0	22.97b	76.787d	0.300a	45.820a	554.143a	0.083b
M3B1	24.05a	91.940b	0.263b	48.610a	473.060b	0.103a
M6B1	20.837c	101.971a	0.205c	43.065b	492.120b	0.088b
M3B3	19.627c	84.133c	0.233c	47.180a	451.240b	0.107a
M6B3	22.403b	97.060b	0.257b	46.445a	469.097b	0.099a

Note: Different letters in the same column indicate significant differences among treatments at the 0.05 level.

## Discussion

The results showed that the application of organic fertilizer could improve soil enzyme activity, soil microbial population structure, root respiration rate, and total root length, root surface area, and root volume significantly increased. This may be due to the application of manure to soils providing additional nutrients that facilitate plant growth^27^, and cotton in the bud stage needs a larger root system to absorb nutrients to maintain growth, and given the poor physical structure of the soil and frequent occurrence of sandstorms, to maintain the supporting functions of the cotton plant. In addition, the stimulating effect of high organic fertilizer (6%) on cotton rhizosphere microorganisms further promoted the effective growth of cotton roots and optimized root architecture^28^.

Biochar has a large specific surface area and strong ionic functional groups due to its highly concentrated C and other nutrients, as well as its porous structure. Through improving soil nutrients, physical and chemical properties, crop root growth, and absorption, crop yield can also be increased^29^. The total root length, total surface area, and Root TTC reducing capacity of cucumber significantly increased with corn straw biochar^30^. Our results showed that organic fertilizer combined with biochar could significantly promote cotton root growth, while maintaining cotton root growth in the later growth stage. Firstly, soil fertility is supplemented by biochar nutrients and trace elements, and secondly, biochar provides living and reproductive space for microorganisms, which improves the structure and abundance of the microbial community^31^. Microorganisms further promote nutrient release of organic fertilizer and biochar^32^. Nutrients mostly exist in organic form in biochar; therefore, the slow degradation process of microorganisms is required, which results in the nutrient release from biochar being slower than that of organic fertilizer, ensuring nutrient supply for the whole growth period. Organic fertilizer combined with low biochar (1%) resulted in the highest cotton root growth, while high biochar (3%) had a negative effect on cotton root growth. It seems that plants are able to take up nutrients with a smaller root system when supplies are optimal, so they have less need of a large root system^33^.

Soil fertility and crop nutrient uptake and utilization efficiency are the main factors restricting crop yield, and efficient nutrient uptake is inseparable from the healthy growth and development of crop roots^34^. Higher Root TTC reducing capacity indicated that the root system was in a healthy soil environment and had a higher metabolic level. Incorporation of organic sources into paddy soil could improve root morphological characteristics and Root TTC reducing capacity of rice plants by increasing root density, active adsorption area, and root surface phosphatase activity^35^. The present study showed that the application of organic fertilizer alone significantly enhanced cotton Root TTC reducing capacity, and owing to the poor nutrient content and physical and chemical properties of grey desert soil, more organic fertilizer was needed to improve the soil environment. Therefore, 6% organic fertilizer treatment resulted in the highest physiological activity of cotton roots.

In addition, the Root TTC reducing capacity of cotton with biochar was higher than that with organic fertilizer alone. The slow release of nutrients from organic manure and biochar provides a healthy soil environment for root survival and activity. It not only provides sufficient raw materials and conditions for root redox reaction, but also adsorbs toxic substances produced in continuous cropping systems^36^. However, in the present study, a high proportion of biochar (3%) could reduce or not affect Root TTC reducing capacity in the bud stage. On the one hand, the growth of cotton roots may be affected by the self-toxicity of aromatic and phenolic substances in high-concentration biochar^37^. On the other hand, biochar and soil have high C/N ratio under high-concentration biochar, which affects the transformation and availability of soil organic C and N, and reduces the availability of soil N^38^, affecting physiological activities such as root respiration.

The results showed that organic fertilizer combined with 1% biochar significantly increased the content of total P and total K in cotton field in the harvesting stage. The correlation analysis showed that the Root TTC reducing capacity of cotton was positively correlated with total K in the bud stage and negatively correlated in the harvesting stage. K may be the key nutrient for physiological activity of cotton roots in this region. The growth and physiological activity of cotton roots peaked in bud stage, and higher K content promoted metabolic activity in this stage. Conversely, owing to the aging damage of cotton roots during harvesting, the roots were fragile. At the same time, the high concentration of K ions in soil destroyed the balance of internal and external ions, which affected the normal physiology of cotton roots and further affected the uptake of ions and nutrients in soil solutes^39^.

The results showed that under the same soil fertility conditions, the roots of high nutrient uptake and utilization crops had root architecture and enzyme activity beneficial to nutrient uptake and transformation^40,41^. NR and GS are the key enzymes for N uptake. The activity of NR and GS can reflect the efficiency of N uptake and utilization by crops^42,43^. The results showed that the application of organic fertilizer increased the activity of NR and GS in cotton roots. Owing to the different roles and functions of the two enzymes in the process of N uptake, there are differences in their performance in the different growth stages^44,45^. In the early stage of cotton growth, N and other nutrients are mainly absorbed, while in the late stage of cotton growth, efficient transformation and transport efficiency, as well as the efficient synthesis of amino acids, are needed to ensure the formation of cotton fibre^46,47^. At the same time, NR is an inducible enzyme, because the application frequency and amount of fertilizer in early growth stage of cotton are higher than that in late growth stage, the NR activity in the bud stage is higher than that in harvesting stage.

The highest root GS activity was in treatments with 1% and 3% biochar in the bud and harvesting stages, respectively. The low amount of biochar added in the early growth stage was enough to meet the growth needs of cotton, while in the late growth stage, more nutrients are needed. The NR activity of cotton roots did not change significantly in the bud stage but was significantly higher in the harvesting stage, indicating that the application of organic fertilizer and biochar played an important role in the nutrient absorption of cotton in the later growth stage.

In addition, the correlation analysis showed that the physiological activity of cotton roots was closely related to soil nutrient status caused by reduction. Yang *et al*.^48^ showed that when the C/N ratio of organic materials was between 20 and 40, the C/N cycle of flue-cured tobacco was more coordinated. Among them, the NR activity and free amino acid content of 20 treatment were significantly higher than those of other treatments in the middle and long term, which was conducive to the accumulation of dry matter. This study showed that the application of organic fertilizer combined with biochar could effectively improve the activity of NR in cotton roots, possibly due to the C/N ratio of organic fertilizer combined with biochar suitable for cotton growth, which could effectively improve the nutrient uptake and utilization efficiency of cotton roots^49^.

Previous studies have reported that organic manure increases plant crop growth and yield in cultivated fields by regulating root morphology and physiological plasticity^50^. The compost added to the greenhouse medium stimulated the rooting of geranium cuttings significantly, increasing their root length by almost 80% and root biomass by over 40%. It also affected a 35% increase in shoot biomass^51^. Gou *et al*.^52^ showed that peanut shell biochar could significantly increase the main root length, diameter, total surface area, and total root density of tomato, and the main root diameter was the main factor causing the yield increase. The middle level of biochar addition(1%) was the most significant factor for yield improvement. In addition, the results showed that proper application of organic fertilizer could effectively improve the physiological activity of cotton root systems, promote N assimilation, and improve cotton yield and fibre quality^53^. The results showed that root architecture not only affected the absorption and utilization efficiency of N and K in soil but also affected the content of N and K in tobacco leaves, thus affecting the quality of tobacco leaves^54^. This study also showed that an increase in soil nutrient content, especially total N content, stimulated the activity of root-related enzymes, promoted the elongation of cotton roots, and established a fine-root architecture. Better root morphological characteristics or strong metabolic activity can affect soil microenvironment around roots by root exudates, which promote cotton roots adaptation to soil environment and effective absorption and utilization of soil nutrients. Cotton yield was positively correlated with the activity of N conversion and absorption enzymes in roots. Therefore, better root management is a fundamental measure to improve cotton yield.

The results of Asai *et al*.^55^ showed that rice yield increased with the increase in biochar application rate after the combination of biochar and N fertilizer. The rice yield of Qu *et al*.^56^ in Hunan and Jiangxi showed that there was no difference in rice yield when biochar was applied alone. The rice yield was significantly higher when biochar was combined with N fertilizer. However, the results of Steiner *et al*.^57^ showed that compared with the treatment without biochar, the dry matter weight of crops treated with biochar decreased by 31.2% and the yield by 56.3% after applying N fertilizer. From the above results, the key to whether biochar can promote crop growth and increase yield is the combination and proportion of fertilizer and biochar and, such as C/N ratio^58,59^. Therefore, when applying biochar, soil fertility should be fully considered, and an appropriate C/N ratio should be used^60^.

In this study, the application of organic fertilizer and biochar alone increased the C/N ratio of cotton field soil, promoted the increase in cotton biomass and cotton seed cotton yield. However, compared with 3% biochar, 1% biochar combined with organic fertilizer had the highest cotton yield. The C content of biochar is more than 50%; therefore, excessive addition (such as 3%) will affect the transformation and availability of organic C and N in soil^61^, which is not conducive to the improvement of cotton yield. Moreover, there is a higher economic benefit from using organic fertilizers combined with 1% biochar.

## Conclusion

In the present study, the combination of organic fertilizer and biochar could alleviate the issues associated with of continuous cropping of cotton fields by improving soil nutrient status, improving cotton root physiology, and optimizing root morphology. The application of organic fertilizer had a significantly positive effect on the growth and yield of cotton, and the higher concentration of organic fertilizer (6%) had a more significant effect on the growth, physiological activity, and yield of cotton roots. Notably, the lower concentration of biochar (1%) promoted cotton root growth and improved root physiological activity. The physiological activity of cotton roots was inhibited when high concentrations of biochar (3%) were added. The results showed that the activity of cotton roots, GS, and NR decreased. In addition, compared with the application of organic fertilizer alone, the increase in cotton yield was more significant by applying biochar, but the amount of biochar should be appropriate.

## Materials and Methods

### Study site and soil collection

**Figure 5:**
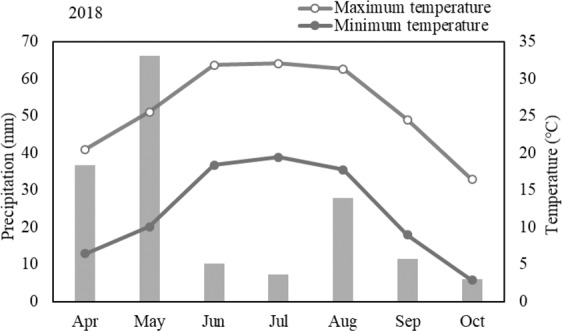
Monthly average temperature and monthly average precipitation at the study site in 2018.

The experiment was conducted from April to October 2018 at Shihezi University Test Site (86 °00′E, 44 °33′N, 442 m above sea level), which is located in Xinjiang Province of Northwest China. The station belongs to temperate continental climate where 7.0 °C remains to be the average air temperature on yearly basis whereas in April to October, the mean maximum temperature is 26 °C, and the mean minimum temperature is 11 °C. The annual precipitation, annual evaporation, and annual sunshine duration are 210.6 mm, 1 664.1 mm, and 2 861.2 h, respectively. And the frost-free period usually lasts 170 d (Fig. 5).

The tested soils were collected from cotton fields in Shihezi University Test Site, which had been cropped for 28 consecutive years and had serious continuous cropping obstacles. Soil samples were collected from the experiment field at 0–30 cm soil depth and were thoroughly mixed to make a representative composite soil sample. The soil used in this study was Grey desert soil that is loess-like diluvial-alluvial, partly Aeolian and slope sediments. It is a kind of soil type with less gravel and more fine grains in desert. The soil used in this study had a soil organic matter (SOM), total N, P, and K content of 28.57 g kg^−1^, 1.25 g kg^−1^, 1.56 g kg^−1^ and 44.80 g kg^−1^, respectively, and an available N, P, and K content of 45.97 mg kg^−1^, 46.77 mg kg^−1^and 649.16 mg kg^−1^.

### Plant materials and fertilization treatments

The organic manure is chicken manure that had a SOM, total N, P, and K content of 235.2 g kg^−1^, 17.8 g kg^−1^, 13.7 g kg^−1^ and 21.8 g kg^−1^, respectively. The tested biochar produced from cotton straw collected from experimental field. The cotton straw was dried at room temperature, and crushed mechanically. Temperature achieved with biochar product at 450 °C with residence time of 6 h. Briefly, the biochar had a pH of 10.04 and contained 625 g kg^−1^ SOC, 0.89 g kg^−1^ TN, 6.9 g kg^−1^ TP, and 23.7 g kg^−1^ TK. The biochar was passed through a 2 mm sieve before experiment.

*cv.Xinluzao* 45 of cotton (*Gossypium hirsutum* L.), a widely planted cultivar in Xinjiang China, was used in the experiment. The field soil was amended with organic manure at 3 rate of 0%, 3%, 6% of the soil dry weight (DW) and combined with rate of 0%, 1%, 3% of biochar. Two growth periods (bud stage and harvesting stage) in the experiment and each treatment was replicated 4 times. Before experiment, the PVC pipe with a height of 40 cm and an inner diameter of 20 cm was buried vertically in the cotton field. The soil, organic manure, biochar and basic fertilizer should be fully mixed. According to the bulk density of 1.30 g cm^−3^, and per pipe with the air-dried soil 15 kg. Except for the difference of treatments, all fertilizers applied in cotton growth period were N 440 kg hm^−2^, P_2_O_5_ 420 kg hm^−2^ and K_2_O 270 kg hm^−2^. Specially, 20% N, 70% P_2_O_5_ and 100% K_2_O of total fertilizers were applied once as a basal. The plants were watered by a drip irrigation system. Residual nitrogen fertilizer was used as topdressing fertilizer, and was applied with water drip in five times during cotton growth period, accounting for 15%, 15%, 20%, 20% and 10% of the total amount of fertilizer applied, respectively. During the whole growth period of cotton, 9 irrigation times were carried out, and the total amount of irrigation was 520 mm. Irrigation and fertilization by drip irrigation under mulch. Cotton was sown on April 30, 2018, with 10 seeds per pot. One seedling with healthy and similar growth were retained in each pot at the time of two leaves and one core. Standard cultural practices were performed during the experiment (i.e., weeding, hoeing, irrigation, etc.), in order to produce healthy cotton.

### Physical and chemical properties of soil measurement

At bud stage (65 days, the period from bud emergence to flowering is called bud stage, and 50% of cotton plants reach the standard of bud emergence) and harvesting stage (135 days, from the beginning to the end of the harvest, with 50% of the bolls as the standard) a mixed soil sample was taken from 5 randomly selected points within each pot. The soils were air-dried and ground to pass through a 1 mm sieve and stored for analyses. Soil organic matter and total N were determined by the dry combustion method at a temperature of 950 °C in the presence of pure oxygen and by the semimicro Kjeldahl method^62^, respectively. The contents of TP, and TK of soil samples were measured by the molybdate colorimetric method after perchloric acid digestion, and the flame photometry method after melting with sodium hydroxide, respectively^62^.

### Root physiology measurement

The bud and harvest stages are important periods in cotton planting. The bud stage is the transition period from vegetative to reproductive growth. The function of the root system plays an important role, which directly affects the later yield. The harvest stage is the overlapping time of vegetative and reproductive growth. It is the key period of cotton yield and fiber development. The growth strategy of the cotton aboveground part and root system is important for the formation of final yield. At bud stage (65 days) and harvesting stage (135 days), all plants in each pot were collected and separated into roots and culms. The root samples were shifted to the laboratory in an ice box within 4 hours. The individual samples were cleaned with de-ionized water (4 °C) to remove residual soil particles and stored in a refrigerator. Root TTC reducing capacity, glutamine synthetase activity (GS) and nirate reductase activity (NR) were determined as described by Li^63^.

### Root morphology measurement

Roots were scanned by scanning (Epson Expression/STD 1600 Scanner) at 800 dpi, and analyzed by winRHIZO root analysis system (Canada Regent Company), in order to measure the Average Root Diameter (ARD), Root Surface Area (RSA), Total Root volume (TRV) and Total Root Length (TRL).

### The dry matter accumulation and yield of cotton

Root dry mass (RDM) and shoot dry mass (SDM) were determined after drying at 105 °C for 30 min and residence temperature at 70 °C to constant weight. Root-shoot ratios were determined by dividing root biomass by shoot biomass. At harvest, 20 plants in the central row of each plot were harvested to measure seed cotton yield and yield components (number of bolls and seed cotton weight per plant). The seed cotton yield was calculated as the seed cotton weight per plant × number of bolls per plant × plant density.

### Statistical analysis

The experiment was conducted in a randomised complete block design, with split-plot arrangements so that the effects of biochar on physical and chemical properties of soil, root physiology, root morphology, and dry matter accumulation of cotton could be tested. The data collected was analyzed using two-way analysis of variance (ANOVA) with the software package SPSS 19.0 (Analytical software, IBM, USA). Means were compared based on the least significant difference test (LSD) at the 0.05 probability level.
